# Long intergenic noncoding RNA01134 accelerates hepatocellular carcinoma progression by sponging microRNA-4784 and downregulating structure specific recognition protein 1

**DOI:** 10.1080/21655979.2020.1818508

**Published:** 2020-09-24

**Authors:** Shiyang Zheng, Yan Guo, Lizhen Dai, Ziming Liang, Qing Yang, Shuhong Yi

**Affiliations:** a Department of thyroid and breast surgery, The Third Affiliated Hospital of Sun Yat-sen University, Guangzhou, China; b Department of breast surgery, The Third Affiliated Hospital of Guangzhou medical college, Guangzhou, China; c Department of Obstetrics, The Third Affiliated Hospital of Sun Yat-sen University, Guangzhou, China; d Department of Hepatic Surgery and Liver Transplantation Center, The Third Affiliated Hospital of Sun Yat-sen University, Guangzhou, China

**Keywords:** Hepatocellular carcinoma, Long noncoding RNA, LINC01134, miR‐4784, SSRP1

## Abstract

Dysregulation of long noncoding RNAs (lncRNAs) has been suggested to foster the carcinogenesis of hepatocellular carcinoma (HCC). To date, the role of long intergenic noncoding RNA01134 (LINC01134) in HCC have never been researched yet. Herein, we found that LINC01134 was highly expressed in HCC tissues in comparison with the matched normal liver tissues and increased LINC01134 expression correlated with shorter overall survival of patients with HCC. Additionally, we demonstrated LINC01134 downregulation significantly suppressed the proliferation ability and colony formation capacity of HCC cells. Furthermore, we revealed that LINC01134 functioned as a competitive endogenous RNA (ceRNA) for miR-4784 to upregulate structure-specific recognition protein 1 (SSRP1) in HCC cells. Meanwhile, miR-4784 inhibitor or restoration of SSRP1 could markedly attenuate the inhibitory effect of LINC01134 downregulation on HCC cells. Taken together, LINC01134 may promote the carcinogenesis of HCC at least partly via the miR-4784/SSRP1 axis. Therefore, LINC01134/miR-4784/SSRP1 axis should be developed as the promising therapeutic target for HCC.

## Graphical Abstract

**Figure:**
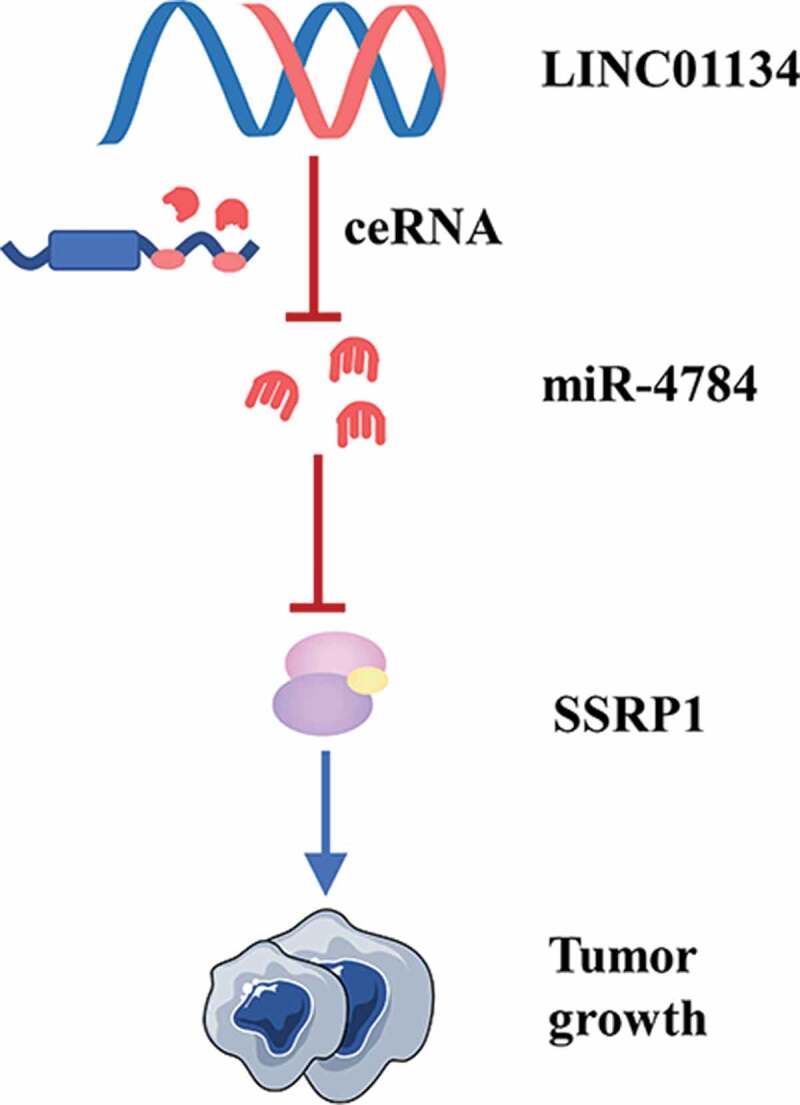

## Introduction

Hepatocellular carcinoma (HCC) is one of the most common malignancies and causes more than 700 000 deaths per year worldwide [1,2]. Although the past decades has witnessed tremendous progress in therapeutic technologies, the HCC patients, especially for those in intermediate and advanced stage, still have a rather poor long-term outcome [3]. Lack of beneficial biomarkers for early diagnosis has been extensively proposed as a key factor resulting in the delayed diagnosis of HCC. Worse still, HCC usually exhibits strong resistance to various therapies due to the complexity of its pathophysiological properties. Therefore, it is of much significance to capture a comprehensive knowledge of the pathological mechanisms for HCC progression, by which we may identify the biomarkers of early diagnosis and therapeutic targets for HCC.

Long noncoding RNAs (lncRNAs) are a class of RNA chains containing more than 200 nucleotides, which lack the capacity of encoding proteins [4]. More and more studies suggest that lncRNAs play important roles in the carcinogenesis of HCC [5]. For instance, Lin et al. found that lncRNA TUG1 was highly expressed in HCC tissues and there was an inverse association between lncRNA TUG1 expression and clinical outcome of patients [6]. Furthermore, they revealed that lncRNA TUG1 could enhance the metastasis of HCC [6]. Wang et al. verified that the amplification of lncRNA UCID gene was frequently enhanced in HCC and lncRNA UCID had a strong promotion on HCC cell G1/S transition and proliferation[7]. Recent study suggested that lncRNA PDIA3P1 was highly expressed in multiple malignant tumors and increased lncRNA PDIA3P1 expression level was related to worse recurrence-free survival of HCC [8]. Interestingly, it was observed that doxorubicin, a typical DNA-damaging chemotherapeutic drug, significantly up-regulated lncRNA PDIA3P1 level in HCC cells. Further investigation demonstrated that lncRNA PDIA3P1 was a crucial driver for the resistance of HCC to doxorubicin [8]. Growing evidence supports that liver cancer stem cell is a key contributor to chemotherapy resistance and tumor recurrence [9]. Coincidently, many lncRNAs, such as lncRNA HAND2-AS1 [10], lncRNA DILC [11], and lncRNA TCF7 [12] have been proposed to modulate the expansion and self-renewal of liver cancer stem cells. Taken together, these studies suggested that lncRNAs may be important candidates for exploring biomarkers applied to early diagnosis and individual treatments of HCC.

Although many a study has identified a body of HCC-associated lncRNA involved in HCC, a comprehensive understanding of lncRNA-mediated carcinogenesis of HCC has never been obtained yet. Long intergenic noncoding RNA01134 (LINC01134) is a identified recently lncRNA, of which the functions in HCC have never been researched yet. Hence, in the current research we first attempted to examine the expression profile and explore the function of LINC01134 in HCC. It has been well established that lncRNAs expressed in the cytoplasm can serve as competitive endogenous RNAs (ceRNAs) to sponge miRNAs, by which they negatively regulate the translation of mRNAs. Of note, increasing evidence suggests lncRNA-miRNA-mRNA regulatory axis is involved in in the carcinogenesis of multiple tumor types including HCC. Therefore, in addition to the biological functions, herein we also further explored whether LINC01134 exerts the potential regulatory effects on HCC cells via the miRNA-mRNA axis.

## Method

### Ethics statement

The ethic committee of The Third Affiliated Hospital of Sun Yat-sen University have approved this study.

### Microarray‐based gene expression analysis

TCGA database (https://cancergenome.nih.gov/) was used to retrieved HCC‐related gene and miRNA. RNA22 database (https://cm.jefferson.edu/rna22/Precomputed/) was used to predict the LINC01134-targeted miRNAs. StarBase database (http://starbase.sysu.edu.cn/) was employed to predict the target of miR-4784.

### Clinical tissues collection

A total of 23 pairs of malignant tissue samples and the matched normal tissue samples were obtained from HCC patients undergoing hepatectomy at The Third Affiliated Hospital of Sun Yat-sen University. All the patients were firstly pathologically diagnosed with HCC. Furthermore, these patients were not treated with chemotherapy, radiotherapy or immunotherapy prior to surgical intervention. The collected samples were quickly frozen in liquid nitrogen once resected and then kept at −80°C.

### Cell culture

Human hepatic cell line L‐02 and HCC cell lines (Huh-7, HepG2, SMCC7721, and Hep3B2) were ordered from the Institute of Biochemistry and Cell Biology of the Chinese Academy of Sciences (Shanghai, China). These cells were grown in Roswell Park Memorial Institute (RPMI) 1640 medium, into which 10% fetal bovine serum (FBS) was added, at 37°C in a humidified 5% CO2 atmosphere.

### Oligonucleotides, plasmids, and cell transfection

Small interfering RNA (siRNA) for negative control (NC) (si-NC) and siRNA against LINC01134 were synthesized by Guangzhou Ribobio Co., Ltd. (Guangzhou, China). Genechem (Shanghai, China) helped to design and synthesize the NC miRNA mimics (miR-NC), miR-4784 mimics, NC inhibitor, and miR-4784 inhibitor. Tumor cells were inoculated in 6-well plates. When the cell confluence covered the 70%‐80% area of plates, transfection was performed using Lipofectamine 2000 (Invitrogen; Thermo Fisher Scientific, Inc., Waltham, MA, USA).

### RNA isolation and real-time quantitative polymerase chain reaction (RT‐qPCR)

Total RNA was extracted from the tissues or cultured cells using TRIzol reagent (Invitrogen). The reverse transcription of the extracted RNA into cDNA was fulfilled using Prime Script RT reagent Kit (Takara, Bio, Japan). SYBR Green method on Roche LightCycler® 480 PCR system was employed to perform RT‐qPCR. GAPDH was regarded as the endogenous control. 2^−ΔΔCt^ method was selected to analyzed all data.

### Ethynyl deoxyuridine (EdU) incorporation assay

EdU incorporation assays were conducted using the EdU kit (Roche, Indianapolis, IN, USA) according to the manufacturer’s protocols. We quantified the fluorescent cells through counting at least five random fields. With the help of the fluorescence microscope (Carl Zeiss, Oberkochen, Germany).

### Colony formation assay

At 24h post-transfection, HCC cells were collected and kept in 6-well plates (3000 cells per well). The cells were incubated with complete medium at 37°C with 5% CO_2_ for 2 14 days. On day 15, tumor cells were fixed in 4% paraformaldehyde and stained with 0.5% crystal violet. At last, the colonies of HCC cells were counted using microscope.

### Nuclear/cytoplasmic fractionation

The PARIS Kit (Invitrogen; Thermo Fisher Scientific, Inc.) was used for detecting the cytoplasmic and nuclear fractions.

### RNA immunoprecipitation (RIP) assay

Magna RIP RNA-Binding Protein Immuno-precipitation Kit (Millipore Inc., Billerica, MA, USA) was employed to detect the binding between miR-4784 and LINC01134. Briefly, we first lysed HCC cells using RIP buffer. Then, we incubated the cell lysates using the magnetic beads conjugated with a human anti-AGO2 antibody or control IgG (Millipore Inc.). After incubation, we digested the protein from the collected samples with proteinase K and then isolated the total RNA using the Trizol for RT-qPCR analysis.

### Luciferase reporter assay

The full length of LINC01134 and the 3′-UTR fragments of structure-specific recognition protein 1 (SSRP1), which contains the wild-type (Wt) binding site of miR-4784 or its mutant (Mut) binding site, were separately cloned into luciferase reporter vector (GeneCopoeia, Inc, Guangzhou, China). HCC ells were inoculated in 24-well plates and cotransfected with Wt or Mut LINC01134 vector or Wt or Mut SSRP1–3ʹ-UTR vector, and miR-4784 mimic or miR-NC. After 48h, these HCC cells were collected for the measurement of luciferase activity by Luc-Pair™ Luciferase Assay Kit (GeneCopoeia, Inc).

### Western blot

Total protein in HCC cells was extracted by the RIPA kit (R0010, Beijing Solarbio Life Sciences Co., Ltd., Beijing, China). Total protein was separated by 10% sodium dodecyl sulphatepolyacrylamide gels electrophoresis (SDS-PAGE), which then was transferred onto the polyvinylidene difluoride (PVDF) membranes (Millipore Corp., USA). The membranes loaded with protein shaken for blockage 2h with 5% skim milk at room temperature. Subsequently, the PVDF membranes were incubated with anti-SSRP1 polyclonal antibody (ab26212, 1:1500 dilution, Abcam, Cambridge, UK, USA) and mouse against GAPDH antibody (ab128915; 1:1500 dilution; Abcam, Cambridge, UK, USA) overnight at 4°C, respectively. After that, we continued to incubate the PVDF membranes with horseradish peroxidase (HRP)-conjugated anti-mouse IgG secondary antibodies (ab205718; 1:5000 dilution; Abcam, Cambridge, UK, USA) at room temperature for 1h. The Enhanced Chemiluminescence Reagent (Bio-Rad Labora-tories, Hercules, CA, USA) were used to visualize the immunoreactive bands.

### Statistical analysis

All statistical processes were fulfilled using SPSS software version 25.0 (SPSS, Inc., Chicago, IL, USA) and Prism 8.0 (GraphPad Software, USA). The data were displayed in the form of the mean ± standard error. The student`s test was used to analyze the difference between two groups. Additionally, when the differences are affected by two factors, the two-way ANOVA for three or more groups were chosen, after which the Sidak`s multiple comparison test was utilized to conduct the post hoc test. The mean LINC01134 expression in TCGA-LIHC was used as a cut-off value to divided 370 patients into low (n=185) and high (n=185) expression groups. The difference in survival analysis was analyzed using the Kaplan‐Meier method. Pearson chi-square test was employed to evaluate the correlation of miR-4784 level with LINC01134 level or expression level and SSRP1 level in HCC tissues. Statistical significance was defined as *p*<0.05.

## Results

### LINC01134 is aberrantly upregulated in HCC tissues and predicts unfavorable prognosis of patients

**Figure 1.**
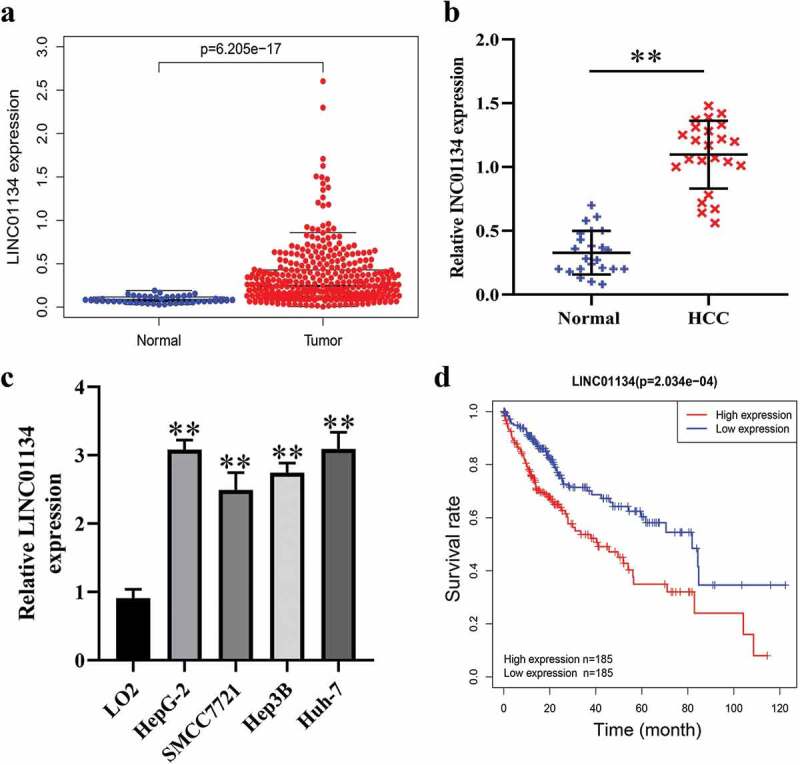
LINC01134 is aberrantly upregulated in HCC tissues and predicts unfavorable prognosis of patients.

We first downloaded data on lncRNA expression profiles in HCC from TCGA database to screen the dysregulated lncRNAs. As result, it was found LINC01134 expression was elevated in tumor tissues compared to normal hepatic tissues (Figure 1(a)). Similarly, by RT-qPCR we also found that LINC01134 was highly expressed in HCC tissues versus the matched noncancerous hepatic tissues from The Third Affiliated Hospital of Sun Yat-sen University (Figure 1(b)). Consistently, LINC01134 was also upregulated in HCC cell lines in comparison with normal human liver cells (Figure 1(c)). Furthermore, bioinformatics analysis based on TCGA data suggested that higher LINC01134 expression correlated with poorer prognosis of HCC patients (Figure 1(d)). Collectively, these results demonstrated that LINC01134 is upregulated in HCC tissues and higher LINC01134 level predicts unfavorable prognosis, implying that LINC01134 may be an oncogene in HCC.

(a) Bioinformatics analysis based on TCGA showed that LINC01134 level was highly expressed in HCC tissues versus adjacent normal liver tissues. (b) RT-qPCR assays showed that LINC01134 expression in HCC tissues from our center was also upregulated versus in adjacent normal liver tissues. (c) RT-qPCR assays showed that LINC01134 level was elevated HCC cell lines (HepG2, SMCC7721, Hep3B2, and Huh-7) compared to normal human liver cells (L-O2). (d) Bioinformatics analysis based on TCGA suggested that higher LINC01134 expression correlated with poor overall survival of HCC patients. **P < 0.01.

### LINC01134 downregulation inhibits the proliferation and colony formation of HCC cells

**Figure 2.**
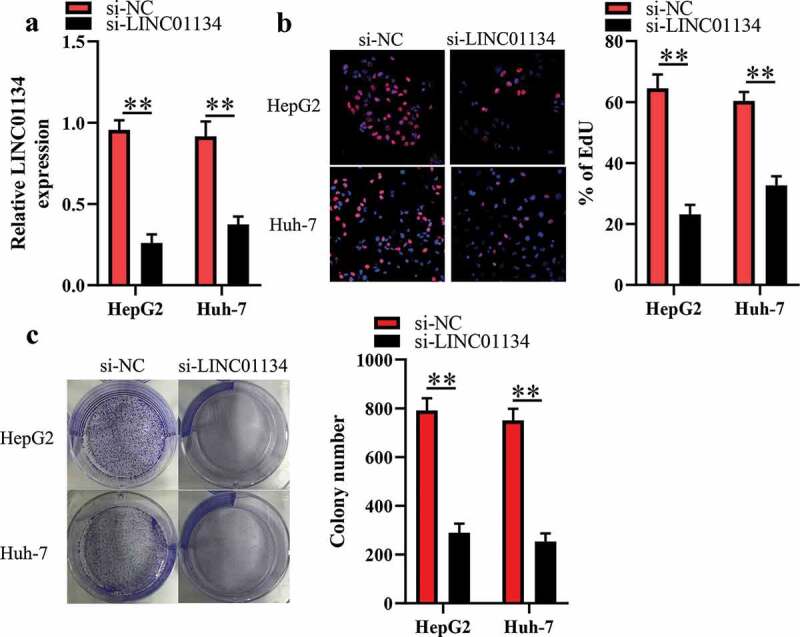
LINC01134 downregulation inhibits the proliferation and colony formation of HCC cells.

HepG2 and Huh-7 cells had higher LINC01134 level than the other HCC cell lines, so we transfected si-LINC01134 or si-NC into the two kind cells and then subjected them for EdU and colony formation assays. As illustrated in Figure 2(a), transfection of si-LINC01134 substantially reduced LINC01134 expression in HCC cells. EdU assays were utilized for evaluating the proliferative ability of HCC cells. We found that transfection of si-LINC01134 significantly decreased the proliferative capacity of HCC cells (Figure 2(b)). Consistently, the colony formation assay showed that si-LINC01134-transfected HCC cells had weaker colony formation capacity than those in si-NC group (Figure 2(c)). Collectively, these results suggested that LINC01134 may function as an oncogene in HCC.

(a) LINC01134 level in HepG2 and Huh-7 cells was significantly reduced after transfection of siRNA against LINC01134 (si-LINC01134) compared to those transfected with si-NC. (b) Transfection of si-LINC01134 markedly inhibited the proliferation of HCC cells versus si-NC. (c) Transfection of si-LINC01134 obviously repressed the colony formation capacity than si-NC.

### LINC01134 works as competitive endogenous RNA (ceRNA) for miR-4784 in HCC cells

**Figure 3.**
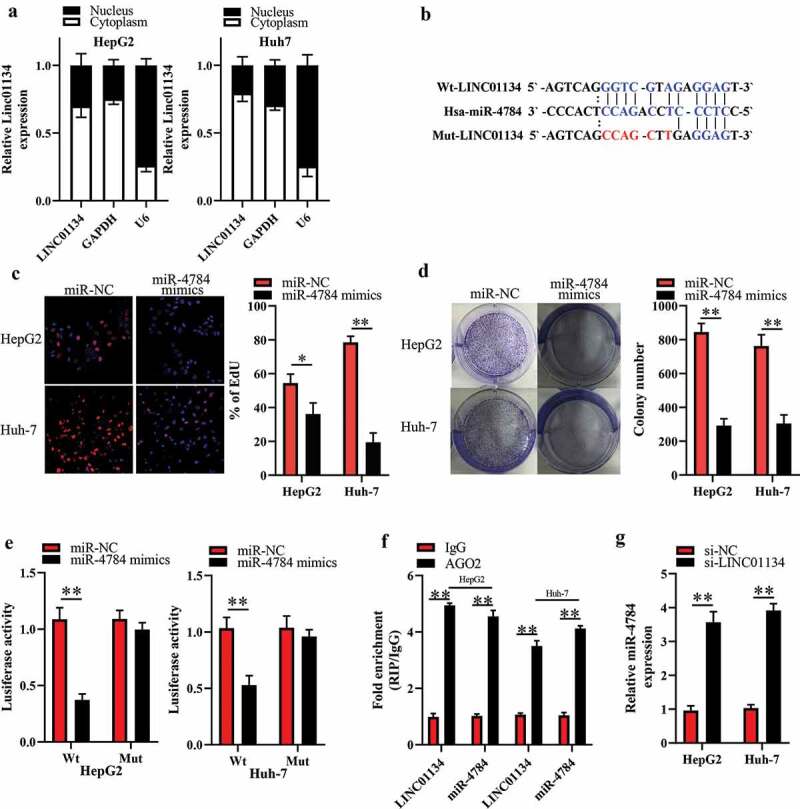
LINC01134 functions as competitive endogenous RNA (ceRNA) for miR-4784 in HCC cells.

To explore the mechanism for the impact of si-LINC01134 on HCC cells, we first detected the subcellular distribution of LINC01134 by nuclear/cytoplasmic fractionation assay. As a result, we observed that LINC01134 was primarily located in the cytoplasm of HCC cells (Figure 3(a)). Cytoplasmic lncRNAs can function as ceRNAs to sponge miRNAs [13–15]. Therefore, we employed the bioinformatics tool (starBase 3.0) to predict the potential LINC01134-targeted miRNAs. A shown in Figure 3(b), there was a potential direct binding between LINC01134 and miR-4784. Interestingly, we found that miR-4784 was downregulated in HCC tissues compared to the adjacent liver tissues (Supplement 1A). Meanwhile, an inverse relationship between LINC01134 and miR-4784 levels in human HCC tissues was verified by spearman’s correlation analysis (Supplement 1B). More importantly, our EdU and colony formation experiments showed that miR-4784 mimics significantly repressed the proliferation (Figure 3(c)) and colony formation (Figure 3(d)) of HCC cells. These results preliminarily dropped a hint that LINC01134 may exert oncogenic function in HCC as a ceRNA for miR-4784. Thus, we performed further experiments to ascertain whether LINC01134 can sponge miR-4784 in HCC cells. As expected, our luciferase reporter assay showed that transfection of miR-4784 mimics dramatically reduced the luciferase activity of LINC01134-Wt, but had no significant impact on the luciferase activity of LINC01134-Mut versus transfection of miR-NC (Figure 3(e)). LINC01134 and miR-4784 were substantially immunoprecipitated by anti-AGO2 antibody in RIP assays (Figure 3(f)), confirming a direct binding between LINC01134 and miR-4784. Based on these clues, we then explored whether LINC01134 had a regulatory effect on miR-4784. As shown in Figure 2(g), LINC01134 silencing markedly increased miR-4784 level in HCC cells. Taken together, these results verified that LINC01134 could serve as a ceRNA for miR-4784 in HCC cells.

(a) Nuclear/cytoplasmic fractionation assay showed that LINC01134 was mainly expressed in the cytoplasm of HCC cells. U6 was used as positive control for nuclear location, while GAPDH was regarded as positive control for cytoplasmic location. (b) Bioinformatics analysis suggested that LINC01134 contain a putative binding site for miR-4784. (c) Overexpression of miR-4784 significantly suppressed the proliferation of HCC cells. (d) Overexpression of miR-4784 significantly inhibited the colony formation of HCC cells. (e) Transfection of miR-4784 mimics dramatically decreased the luciferase activity of LINC01134-Wt, but had no significant impact on the luciferase activity of LINC01134-Mut versus transfection of miR-NC. (f) LINC01134 and miR-4784 in HCC cells were substantially immunoprecipitated by anti-AGO2 antibody in RIP assays. (g) Silencing LINC01134 markedly increased miR-4784 level in HCC cells. *P < 0.05. **P < 0.01.

### miR-4784 performs tumor-suppressive function by targeting SSRP1 in HCC cells

**Figure 4.**
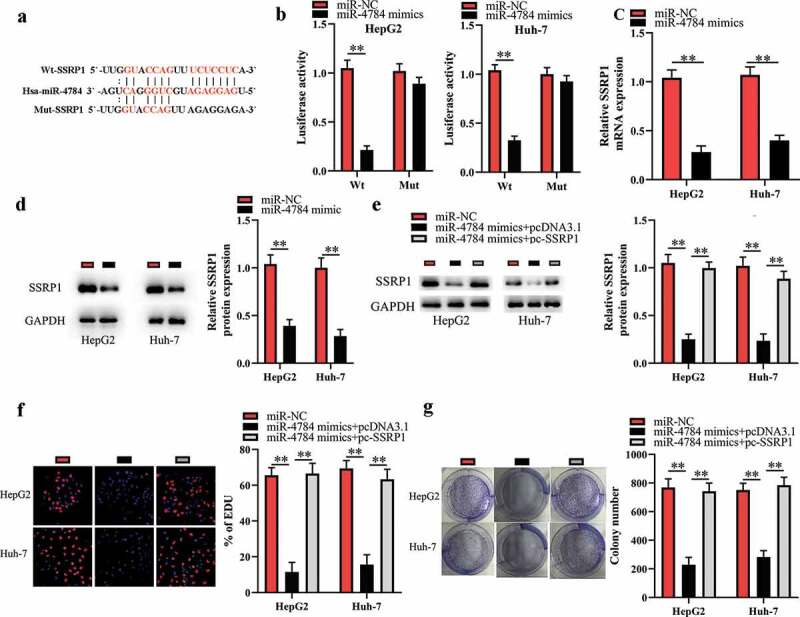
miR-4784 performs tumor-suppressive function by targeting SSRP1 in HCC cells.

The bioinformatics analysis indicated that SSRP1 mRNA might be a potential target of miR-4784 (Figure 4(a)). Of interest, several studies have showed that SSRP1 acts as an oncogene in multiple malignancies including HCC [16–18]. Therefore, we hypothesized that miR-4784 inhibited the proliferation and colony formation of HCC cells by impeding the translation of SSRP1 mRNA. To verify this hypothesis, we conducted the luciferase reporter assays to further explore the direct interaction between miR-4784 and SSRP1 mRNA. As a result, transfection of miR-4784 mimics reduced the luciferase activity of SSPR1-Wt, while transfection of miR-4784 mimics did not alter the luciferase activity of SSRP1-Mut in HCC cells (Figure 4(b)), confirming a direct interaction between miR-4784 and SSRP1 mRNA. In accordance with this, our further investigation demonstrated that miR-4784 overexpression dramatically reduced SSRP1 mRNA (Figure 4(c)) and protein levels (Figure 4(d)) in HCC cells. Meanwhile, spearman’s correlation analysis uncovered that miR-4784 level was inversely associated with SSRP1 mRNA level in human HCC tissues (Supplement 2). In general, these results indicated that miR-4784 could negatively regulate SSRP1 in HCC cells. Cotransfection of pc-SSRP1 markedly reversed the effect of miR-4784 mimics on SSRP1 expression (Figure 4(e)). More importantly, restoration of SSRP1 expression significantly abrogated the suppressive effect of miR-4784 mimics on the proliferation (Figure 4(f)) and colony formation of HCC cells (Figure 4(g)). Taken together, these data suggested that miR-4784 represses the carcinogenesis of HCC by downregulating SSRP1 expression.

(a) Bioinformatics analysis suggested that SSRP1 mRNA was a potential target of miR-4784. (b) Transfection of miR-4784 mimics reduced the luciferase activity of SSPR1-Wt, whereas the luciferase activity of SSRP1-Mut was not altered by transfection of miR-4784 mimics in HCC cells. (c) Overexpression of miR-4784 dramatically reduced SSRP1 mRNA level in HCC cells. (d) Overexpression of miR-4784 dramatically reduced protein level in HCC cells. (e) Cotransfection of pc-SSRP1 markedly reversed the effect of miR-4784 mimics on SSRP1 level. (f) Restoration of SSRP1 expression significantly abrogated the inhibitory effect of miR-4784 mimics on HCC cell proliferation. (g) Restoration of SSRP1 expression significantly abrogated the inhibitory effect of miR-4784 mimics on HCC cell colony formation. **P < 0.01.

### LINC01134 downregulation exerts tumor-suppressive functions through regulating miR-4784/SSRP1 axis in HCC cells

**Figure 5.**
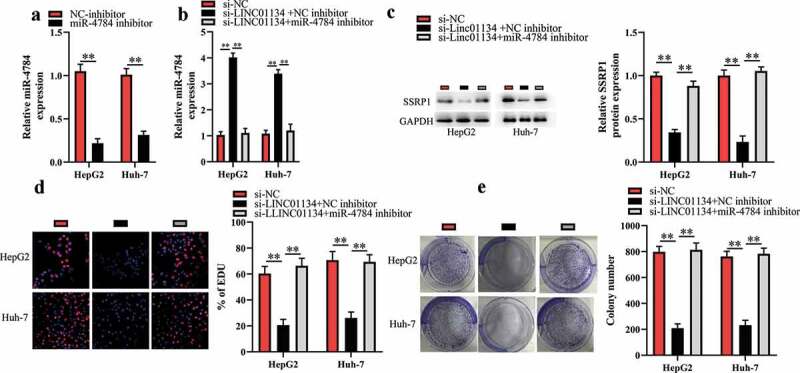
LINC01134 downregulation exerts tumor-suppressive functions through regulating miR-4784/SSRP1 axis in HCC cells.

Based on the aforementioned results, in this part we further investigated whether LINC01134 downregulation repressed the proliferation and colony formation of HCC cells via the miR-4784/SSRP1 pathway. First, HCC cells were cotransfected with siRNA against LINC01134 and miR-4784 inhibitor and then were subjected for subsequent experiments. As shown in Figure 5(a-b), miR-4784 inhibitor markedly reversed the effect of LINC01134 downregulation on miR-4784 level in HCC cells. As expected, the inhibitory effect of LINC01134 downregulation on SSRP1 level could be restored by miR-4784 inhibitor (Figure 5(c)). Consistently, we found that miR-4784 inhibitor significantly abrogated the effect of LINC01134 downregulation on the proliferation (Figure 5(d)) and colony formation (Figure 5(e)) of HCC cells. Taken together, LINC01134 may promote the carcinogenesis of HCC at least partly via the miR-4784/SSRP1 axis.

(a) miR-4784 inhibitor markedly reduced miR-4784 level in HCC cells. (b) miR-4784 inhibitor markedly reversed the effect of LINC01134 downregulation on miR-4784 level in HCC cells. (c) The inhibitory effect of LINC01134 downregulation on SSRP1 level was significantly restored by miR-4784 inhibitor. (d) miR-4784 inhibitor markedly abrogated the effect of LINC01134 downregulation on the proliferation of HCC cells. (e) miR-4784 inhibitor significantly abrogated the effect of LINC01134 downregulation on the colony formation of HCC cells. **P < 0.01.

LINC01134 is upregulated in HCC tissues

High LINC01134 level correlates with poor prognosis of HCC patients

LINC01134 promotes HCC cell proliferation via miR-4784/SSRP1 axis

## Discussion

Although huge progress in diagnostic and therapeutic technologies has been made during the past decades, the patients with HCC, especially for those in intermediate and advanced stage, still have a rather dismal prognosis [3]. Thus, it is of much significance to comprehensively understand the pathological mechanisms for the carcinogenesis of HCC, in order to find beneficial biomarkers for early diagnosis and therapeutic targets of HCC. In this study, we found that LINC01134 was highly expressed in human HCC tissues and cell lines. Furthermore, higher LINC01134 expression level correlated with poor prognosis of patients with HCC. More importantly, we demonstrated that LINC01134 downregulation significantly inhibited the proliferation and colony formation of HCC cells. Collectively, these results suggested that LINC01134 may act as an oncogene in HCC.

It has been well established that cytoplasmic lncRNAs perform various physiological and pathological functions mainly by acting as ceRNA for miRNAs [13–15]. Herein, we found that LINC01134 was richly expressed in the cytoplasm of HCC cells. This result hinted that LINC01134 may promote the carcinogenesis of HCC as ceRNA for specific miRNAs. As expected, our further investigations demonstrated that LINC01134 could competitively sponge miR-4784 in HCC cells. Dysregulation of miR-4784 has been suggested to be involved in cancer progression. Tao et al. reported that miR-4784 expression was down-regulated in upper urinary tract carcinoma (UUTC) tissues and it exerted tumor-suppressive effects on UUTC by targeting FGFR3 [19]. Feng et al. suggested that downregulation of miR-4784 could increase AHDC1 expression level in cervical cancers, which aggravated the tumor progression [20]. Interestingly, a study by Zhen et al. [21] verified that miR-4784 was downregulated in HCC tissues versus adjacent normal liver tissues, and lower miR-4784 expression level closely correlated with poor prognosis of patients with HCC. However, the functions of miR-4784 in the carcinogenesis of HCC have never been studied. In line with the previous study, in this study we also found that miR-4784 was downregulated in HCC tissues compared to the adjacent noncancerous liver tissues. In addition, we initially demonstrated that miR-4784 could suppress the proliferation and colony formation of HCC cells. Moreover, our data showed that miR-4784 inhibitor markedly reversed the effects of LINC01134 downregulation on HCC cells. Taken together, these results suggested that LINC01134 may promote the carcinogenesis of HCC by sponging miR-4784 as ceRNA.

Solid evidence shows that miRNAs can directly interact with the 3′-UTRs of mRNAs, by which miRNAs inhibit the translation of mRNAs [22,23]. By bioinformatics prediction, we identified structure-specific recognition protein 1 (SSRP1) mRNA as a potential target of miR-4784. Moreover, this result was further validated by the luciferase reporter assays. SSRP1 has been considered as an oncogene in a multiple of human malignancies including HCC [16–18]. Thus, we asked whether miR-4784 functioned as a tumor suppressor by targeting SSRP1 in HCC cells. To answer this question, we first examined the influence of miR-4784 mimics on SSRP1 level in HCC cells. The result showed that miR-4784 mimics significantly reduced the SSRP1 expression level in HCC cells. Meanwhile, we found that restoration of SSRP1 expression markedly reversed the effect of miR-4784 mimics on HCC cells. These results suggested that miR-4784 could inhibit the carcinogenesis of HCC by targeting SSRP1. Interestingly, we found that si-LINC01134-induced decrease in SSRP1 level could be restored by miR-4784 inhibitor. Meanwhile, miR-4784 inhibitor was able to abrogate the effect of si-LINC01134 on HCC cells. Taken together, our findings revealed that LINC01134 may promote the carcinogenesis of HCC at least partly by regulating the miR-4784/SSRP1 axis.

Several limitations existed in our research. First, gain-of-function assays by overexpressing LINC01134 were not performed to further determine the role of LINC01134 in HCC cells. Second, in addition to cell proliferation, we did not evaluate the effects of LINC01134 on the other phenotypes of HCC cells, such as apoptosis, invasion and chemoresistance. At last but not least, the oncogenic role of LINC01134 in HCC was not assessed in vivo model. In future studies, we will resolve these limitations to further validate the oncogenic role of LINC01134 in HCC.

In conclusion, our study showed that LINC01134 was highly expressed in HCC tissues and increased LINC01134 level correlated with unfavorable prognosis of HCC patients. Moreover, we revealed that LINC01134 facilitates the carcinogenesis of HCC by regulating the miR-4784/SSRP1 axis. Therefore, LINC01134/miR-4784/SSRP1 axis may be the promising therapeutic target for HCC.

## Supplementary Material

Supplemental Material

Supplemental Material
