# Supplementary figures and images for: Long intergenic noncoding RNA01134 accelerates hepatocellular carcinoma progression by sponging microRNA-4784 and downregulating structure specific recognition protein 1
Source: Bioengineered. 2020 Sep 24;11(1):1016–26. doi: 10.1080/21655979.2020.1818508 (PMC8291876; doi:10.1080/21655979.2020.1818508)

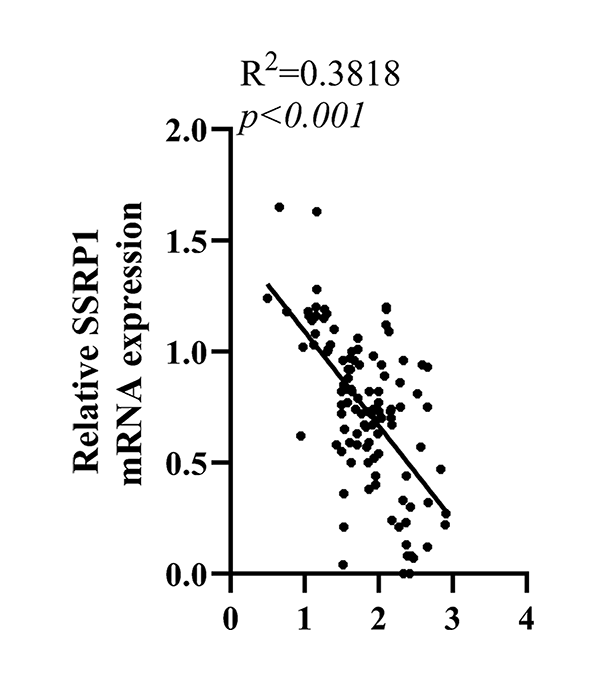

Supplement: Supplemental Material [file KBIE_A_1818508_SM5938.tif]

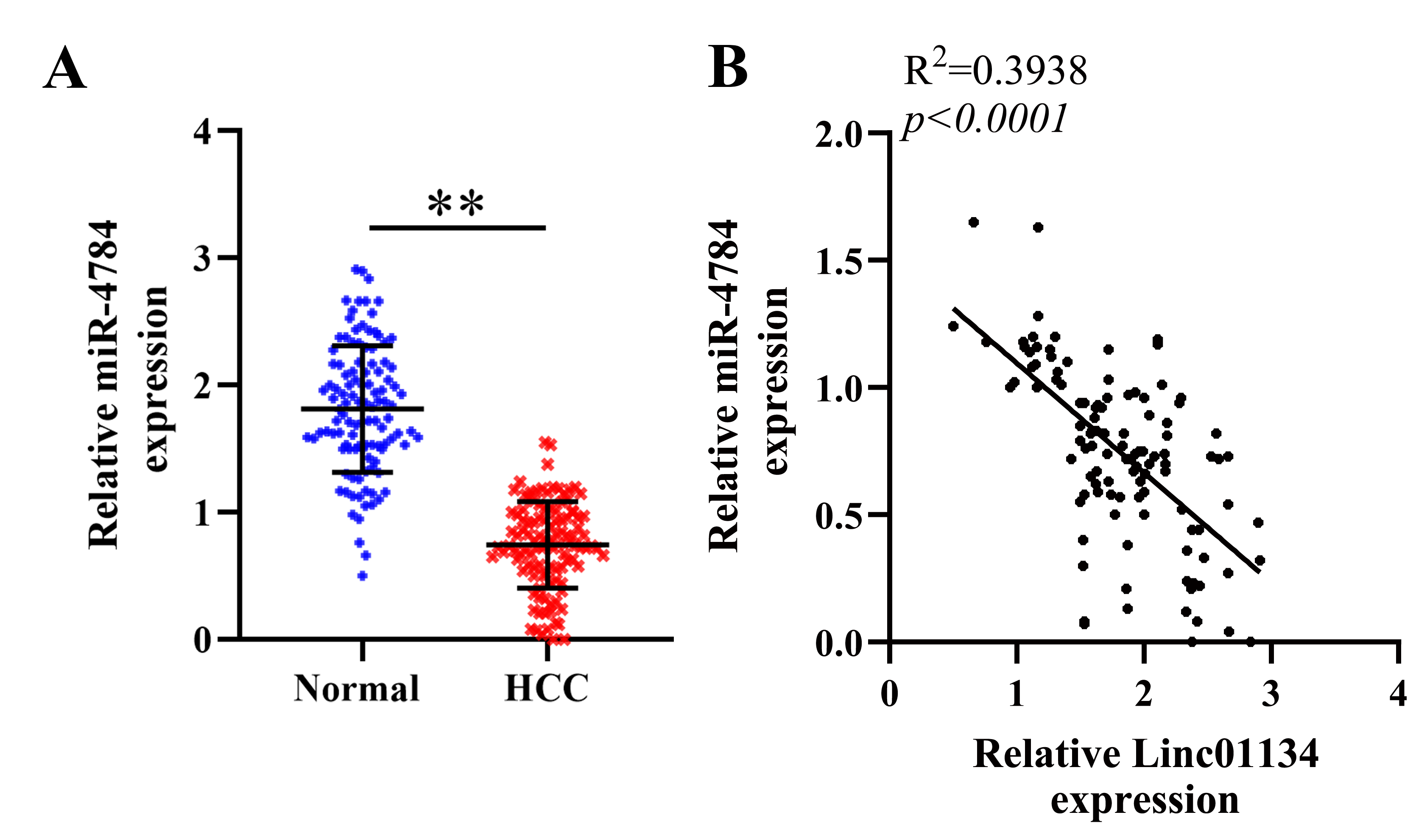

Supplement: Supplemental Material [file KBIE_A_1818508_SM5921.tif]
